# Climate Change and ENSO Effects on Southeastern US Climate Patterns and Maize Yield

**DOI:** 10.1038/srep29777

**Published:** 2016-07-19

**Authors:** Spyridon Mourtzinis, Brenda V. Ortiz, Damianos Damianidis

**Affiliations:** 1 Department of Agronomy, University of Wisconsin-Madison, Madison WI 53706, USA; 2 Department of Crop, Soil & Environmental Sciences, Auburn University, 201 Funchess Hall, Auburn, AL 36849, USA.

## Abstract

Climate change has a strong influence on weather patterns and significantly affects crop yields globally. El Niño Southern Oscillation (ENSO) has a strong influence on the U.S. climate and is related to agricultural production variability. ENSO effects are location-specific and in southeastern U.S. strongly connect with climate variability. When combined with climate change, the effects on growing season climate patterns and crop yields might be greater than expected. In our study, historical monthly precipitation and temperature data were coupled with non-irrigated maize yield data (33–43 years depending on the location) to show a potential yield suppression of ~15% for one °C increase in southeastern U.S. growing season maximum temperature. Yield suppression ranged between −25 and −2% among locations suppressing the southeastern U.S. average yield trend since 1981 by 17 kg ha^−1^year^−1^ (~25%), mainly due to year-to-year June temperature anomalies. Yields varied among ENSO phases from 1971–2013, with greater yields observed during El Niño phase. During La Niña years, maximum June temperatures were higher than Neutral and El Niño, whereas June precipitation was lower than El Niño years. Our data highlight the importance of developing location-specific adaptation strategies quantifying both, climate change and ENSO effects on month-specific growing season climate conditions.

Average global temperatures have increased by 0.4 °C during the last three decades and in some regions even greater increases have been observed^1^. The influence of in-season weather trends on crops yields such as soybean, wheat, and maize has been explored in several countries^2^,^3^,^4^,^5^,^6^,^7^,^8^. Quantified impacts of climate change in a US-wide study showed a 17% potential yield decrease in maize yield for each degree increase in growing season temperature^4^. However, weather-related variability in agricultural production is region-specific and can reach up to 80%^9^. In addition, crop responses to weather fluctuations vary among vegetative and reproductive stages^10^ and the period of time used to quantify climate trends has been identified as an important factor when evaluating the effects of climate on agriculture^5^,^11^. Therefore, the region-specific evaluation of climate change effects on agricultural production based on early-(planting to early vegetative crop growth stages), mid-(late vegetative stages to early reproductive growth stages) and late-growing season (late reproductive growth stages to harvest) climate trends is imperative^2^,^11^.

Examination of temperature anomalies during maize growing season is of great importance as it strongly connects to yield. Temperatures lower than 10 °C greatly retard photosynthesis and development^12^; nevertheless, such low temperatures during the growing season months are not common in most maize-growing areas. Increased maximum temperatures during late vegetative (e.g., tasseling) and early reproductive growth stages (e.g., grain filling) shorten the ripening period, stress the crop, and reduce final yield^13^. Additionally, elevated temperatures increase evapotranspiration rates (evaporation and plant transpiration) which can result in water deficit. The associated yield impact is more apparent in drought events (non-irrigated agriculture) during the one month period before and after the first reproductive growth stage^14^. A few studies have demonstrated the importance of maximum temperatures^15^ and growth stage-specific precipitation amount^11^,^16^,^17^,^18^ on maize yield in West North Central U.S. Therefore, across the southeastern U.S., examination of maximum temperatures and cumulative precipitation in fine temporal resolution, such as monthly rather than seasonal, is important.

The El Niño Southern Oscillation (ENSO) is an ocean-atmospheric phenomenon characterized by inter-annual sea surface temperature, surface air pressure variability, and atmospheric circulation that occur across the equatorial Pacific Ocean^19^,^20^. The ENSO phases have also been related to agricultural production variability^21^,^22^,^23^ as it is accepted that it can affect climate patterns and crop yields in both, the southern^24^,^25^,^26^ and northern hemisphere^27^. The phenomenon has three phases (El Niño, La Niña, and Neutral) and during every ENSO phase the weather conditions in southeastern U.S. vary significantly resulting in high seasonal climate variability. The El Niño phase is characterized by wetter than normal fall to spring period^28^,^29^ and the La Niña phase is characterized by cooler than normal summer months and drier than normal winter and spring weather conditions^30^.

Variation in year-to-year spring and summer climate conditions can be attributed to ENSO condition and to climate change. A few studies have focused on month-specific weather anomalies (mediated by climate change) effects on crop yield^2^,^25^,^26^,^31^. However, the effect of a strong ENSO phase on month-specific climate patterns and its associated effect on a crops’ yield in not well examined. Season-wide effects averaged across large areas is useful information; however, warming trends are not spatially and temporally uniform^32^. Therefore, fine temporal resolution and location-specific quantification of both, climate change and ENSO effects, on climate patterns and crop yield is important for growers and agricultural-related industries since potential yield impacts can result in significant monetary loss and put food security at risk^2^.

## Results and Discussion

We show that over the past 33 years (1981–2013), monthly maximum temperature and precipitation trends varied among locations (Supplementary Material Table S1). The across locations observed non-uniformity is in agreement with previous studies^2^,^32^. Most maximum temperature trends were positive across months and locations and were all positive when averaged across all regions. Nevertheless, differences in magnitude existed among locations. For example at Belle Mina, which located in north Alabama, the maximum temperature trend in June was the highest observed and monthly trends over the period June-September were higher than in all other locations. The precipitation trends were more variable than maximum temperature trends among locations and examined periods. During spring months, precipitation trends were negative whereas most July-September trends were positive. In June, trends varied among the five locations. However, the southeastern U.S. monthly average trends were negative in five out of the seven months of the maize growing season. Flowering and silking are the growth stages with the greatest water demand and occur in June across the examined region. Therefore, the importance of examining the month-specific weather effects on maize yield is further highlighted.

Location-specific climate-yield statistical models based on monthly maximum temperature and monthly cumulative precipitation accounted for 74–91% in maize yield variability. From all the locations, the Blairsville-specific model resulted in non-significant regression (Table 1). In the case of maximum temperature, effects in June were the greatest. According to our estimates, a 1 °C increase in March maximum temperature increased yield in Tifton by 268 kg ha^−1^ and suppressed yield in Fairhope by 760 kg ha^−1^. The same increase in April temperature increased yield in Belle Mina by 600 kg ha^−1^ whereas 1 °C increase in June decreased Belle Mina and Prattville yields by 1262 kg ha^−1^ and by 735 kg ha^−1^ respectively. A 1090 kg ha^−1^ reduction in Belle Mina and a 524 kg ha^−1^ yield reduction in Tifton was associated with 1 °C increase in September and in July, respectively; however, the same increase in September maximum temperature favored yield in Prattville by 609 kg ha^−1^. When aggregated in southeastern U.S. average, the negative effects of maximum June and July temperatures were significant and resulted in 15% maize yield suppression. The weather conditions during these two months, and especially of June, have been identified as key in estimating impacts on maize yield^11^,^17^,^18^. Some variability on which month-specific weather conditions had the greatest effect on maize yield was observed among individual locations. However, this was attributed to topography-microclimate and soil type differences (Supplementary Material Table S2). Nevertheless, across the examined region, the 1981–2013 maximum temperature anomalies during June and July were suppressing yields more than the rest growing season months. The greater impact of these two summer months on maize yield compare to the rest was attributed to the sensitivity of maize to weather conditions the one month period before and after the first reproductive growth stage^14^ (in June and July).

In the case of monthly cumulative precipitation, a 10 mm precipitation increase in March favored maize yield in Tifton by 80 kg ha^−1^. The precipitation increase in April however, suppressed yield in Fairhope by 137 kg ha^−1^. The same increase in May precipitation favored yields in Prattville and in Tifton by 141 and 187 kg ha^−1^, respectively. Similar responses were associated with precipitation increase in June at both locations. An increase in July precipitation increased maize yield in Prattville and decreased yield in Tifton. A 109 kg ha^−1^ yield suppression in Fairhope was associated with increased August precipitation. When yield impacts were averaged across the southeastern locations, increase in cumulative precipitation anomalies in April reduced maize yield, whereas increase in June precipitation contributed to higher yields. The beneficial effect of reduced precipitation in the planting period (March-April) and that of increased precipitation during kernel filling periods (starting in June) has also been documented in a study in central U.S.^11^.

These results reveal the potential significant effects on the in-season weather variability mediated by climate change. Over the 33 years of the study, the potential yield effect of a 1 unit change in both, in-season monthly maximum temperature and cumulative precipitation, was negative and ranged from −25.1 to −1.6% losses depending on the location. When data were aggregated across locations, the climate change contribution resulted in 15.2% maize yield suppression. The most significant contribution was due to a 1 °C increase in June and July maximum temperatures that accounted for ~50% of the yield suppression respectively. These results demonstrate the greater impact of maximum temperature anomalies on maize yield compared to precipitation trends. This result is in agreement with previous reported climate change effects on soybean yield^2^. It appears that increased maximum temperatures, during late vegetative and reproductive crop growth stages, stress the plant which is reflected in significant yield losses. This result is in further agreement with previous studies examining the maize growth stage-specific effect of maximum temperature and precipitation on final yield^11^,^15^,^17^,^18^. Although all the locations of the study are in the southeastern U.S. (Supplementary Material Table S2), the microclimate differences can be significant, especially when moving from the gulf shore (South-Fairhope) to the state boarders with Tennessee (North-Belle Mina). Additionally, soil type differences play an important role in the effect of precipitation on maize yield. For example, silt and clay soil particles (e.g., Belle Mina, Blairsville) exhibit improved water holding capacity, and therefore increased plant available water potential than sand particles (e.g., Prattville, Tifton, Fairhope). Therefore, the beneficial effects of increased precipitation would be more apparent in non-irrigated maize grown at locations with sandy soils. Additionally, moisture abundance in southeastern U.S. might have diminished the magnitude of the observed precipitation effects on maize yield. However, it should be noted that the effects of extreme flooding events have not been examined and therefore, the associated impacts might be greater than the effects we report.

The average annual yield gain across the locations of our study was 70 kg ha^−1^yr^−1^. Month-specific weather anomalies had variable effect on yield trend (Table 2). The greatest yield trend suppression was observed in Belle Mina (−89 kg ha^−1^yr^−1^) due to maximum temperature anomalies in June (+0.07 °C yr^−1^). Similarly, the combined yield trend suppression in Prattville reached 23 and 32.6 kg ha^−1^yr^−1^ due to March-September maximum temperature and precipitation anomalies, respectively. When data was aggregated and averaged across the southeastern U.S., the annual yield gain since 1981 was suppressed by ~25% (17.1 kg ha^−1^yr^−1^), which was mainly influenced by year-to-year June maximum temperature anomalies. Due to the commonly used planting dates in the region, the late vegetative and early reproductive growth stages (when the crop is most susceptible to heat and drought stress) occur in June. These results are in agreement with previous studies^11^,^14^,^17^,^18^ and further highlight the importance of June weather conditions on maize annual yield gain in the southeastern U.S.

Variable month-specific climatic conditions were observed among ENSO phases from 1971–2013 (Supplementary Material Figure S1A−D). Average, minimum, and maximum temperatures were greater during strong La Niña phase in March, April and June by 1.1, 1, and 0.7 °C, respectively, when compared to Neutral and El Niño phases. However, this pattern was reversed in July when 0.4 °C higher temperatures were observed during El Niño years. Large differences in precipitation among ENSO phases were detected in April, June, July, August, and September. Precipitation during El Niño was greater in April by 22 mm, in June by 10 mm and in August by 9.5 mm compared to La Niña phase. However, in strong La Niña years, a greater precipitation amount was observed in July (+17.4 mm) and in September (+32 mm) compared to El Niño years. These differences suggest that the monthly in-season climate patterns in southeastern U.S. vary greatly due to the influence of a strong ENSO condition from December-March.

**Figure 1:**
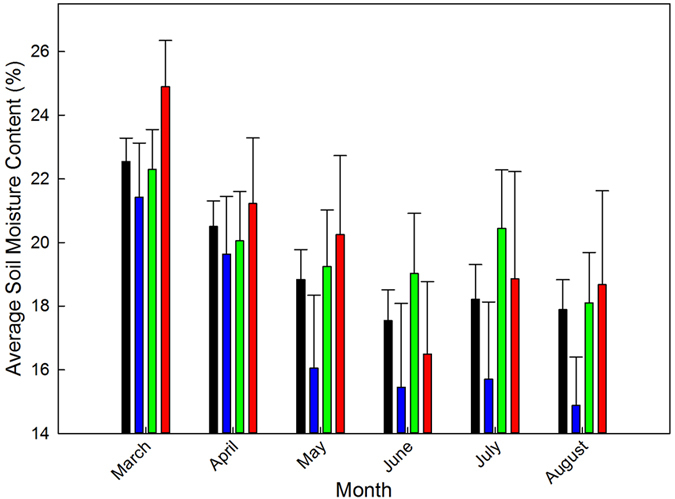
Month-specific soil moisture content (%) averaged across Blairsville, Tifton and Calhoun in GA (2003–2013) by ENSO phase. Bars in black color represent the 11-year average soil moisture content. Bars in blue color represent the average soil moisture content of the La Niña years. Bars in green color represent the average soil moisture content of the Neutral years. Bars in red color represent the average soil moisture content of the El Niño years. Standard errors represent the standard error of the mean.

**Figure 2:**
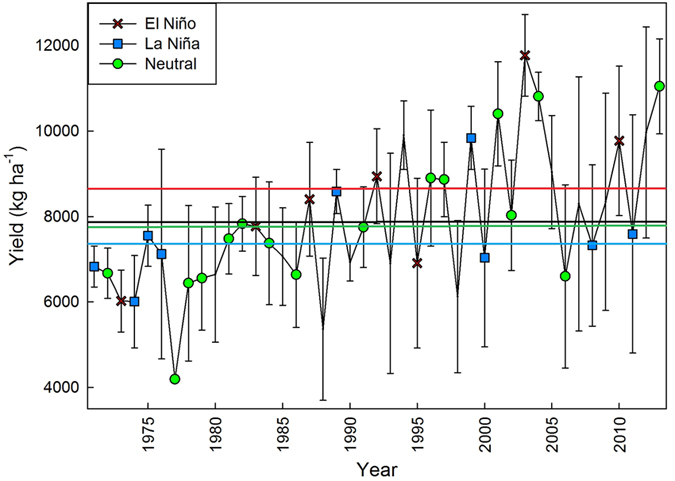
Year-specific maize yield (kg ha^−1^) from 1971–2013 averaged across the locations of the study. Blue rectangles represent yields during La Niña ENSO years. Green circles represent yields during Neutral ENSO years. Red crosses represent yields during El Niño ENSO years. The horizontal black line represents the 43-year yield average (Yield = 7853 kg ha^−1^, s.e.m = 245 kg ha^−1^). The horizontal green line represents the Neutral ENSO years average yield (Yield = 7850 kg ha^−1^, s.e.m = 454 kg ha^−1^). The horizontal blue line represents the La Niña ENSO years average yield (Yield = 7543 kg ha^−1^, s.e.m = 367 kg ha^−1^). The horizontal red line represents the El Niño ENSO years average yield (Yield = 8515 kg ha^−1^, s.e.m = 719 kg ha^−1^). Standard errors represent the standard error of the mean.

The final crop yield is strongly affected by changes in soil moisture content. This relation is strong in southeastern U.S. where most of agricultural land is non-irrigated and soils across the Coastal Plain have low water holding capacity. From 2003 until 2013, that data was available at 3 locations in GA, month-dependent greater soil moisture content was measured during El Niño years when compared to La Niña years by 1–4.2% (Fig. 1). This was justified by examining the month-specific cumulative precipitation during these 11 years across the three locations (Supplementary Material Figure S2). Apart from March, in every month with a strong El Niño phase the precipitation was greater than the same months with a strong La Niña phase. It is interesting that in four out of the seven growing season months, the month-specific cumulative precipitation during neutral years was also greater than La Niña years precipitation which was considered as the main reason for greater soil moisture content in neutral years compared to La Niña years. The influence of the observed differences in temperatures, precipitation, and soil moisture were reflected in final yield (Fig. 2). During the 43 years of the study large year-to-year variations in maize yield were observed. La Niña average yield reached 7500 kg ha^−1^ (range = 3830 kg ha^−1^) whereas El Niño average yield reached 8500 kg ha^−1^ (range = 5750 kg ha^−1^). The yield average of El Niño years was ~1000 kg ha^−1^ greater than La Niña average yield (~12% difference).

As discussed earlier, in southeastern U.S., June weather conditions are of great importance for maize production since late vegetative and early reproductive growth stages occur. The greater precipitation, soil moisture, and lower maximum temperatures during El Niño years compared to La Niña could justify the overall yield differences among ENSO phases. This result further underlines the importance of identifying the ENSO- and month-specific meteorological factors that result in such great yield variability rather than focusing only on ENSO effects or solely on climate change effects on maize yield.

It should be noted that other factors apart from ENSO can also result in changes of precipitation amount, frequency, and intensity. Variability in precipitation events have also been attributed to an increasing hydroclimatic intensity which can lead to shorter and less frequent precipitation events^33^. Global projections of changes in precipitation and aridity (defined as the ratio of total annual precipitation to potential evapotranspiration) have highlighted the risk of land degradation^34^ which can further increase uncertainties associated with agricultural production and food security.

The sensitivity of the results to the analysis used (least squared method) was evaluated by repeating the analysis using the maximum and restricted maximum likelihood methods. The direction and magnitude of the effects did not change and therefore we report the least squared method results. Also, since no extreme weather yield impacts are included in this study, we consider the documented effects on maize yield as a lower bound given that climate change is likely to increase the frequency of extreme weather events^35^ and the associated impacts on crop production and food security^36^.

We show that climate anomalies are month-specific and can have significant negative effect on maize yield and on the annual yield gain. Under average climatic conditions, yields and annual yield trend across the southeastern U.S. might have been greater than those observed during the 43 years of the study. However, apart from the negative effects of climate change, the southeastern U.S. climate is strongly influenced by ENSO condition. Maximum temperatures in June during La Niña years were 0.7 °C higher than El Niño years and cumulative precipitation was 10 mm lower. However, maximum temperatures in July during La Niña years were 0.4 °C lower than El Niño years and cumulative precipitation was 17 mm higher. These results show that month-specific weather patterns vary between Niña and Niño years. According to our estimates of maximum temperature effects on maize yield, a significant suppression might be associated due to elevated maximum June temperatures during La Niña years and due to increased maximum July temperatures during El Niño years.

Adaptation strategies can mitigate climate-induced negative effects on crop yields^37^. The strong connection between southeastern U.S. climate and ENSO phases has resulted in seasonal climate forecast with lead times up to several months prior to the spring planting season in the region^38^. For a given ENSO phase, a grower can use the seasonal climate forecast to select the management practices that reduce the risk of adverse climatic conditions or the ones that will capitalize on the favorable climate conditions. These management practices can include adjusting planting dates to avoid the higher than normally expected temperatures during reproductive growth stages of the crop, or to take advantage of the expected increased spring precipitation as a result of an El Niño phase. Our results suggest that future year-to-year maize yield variability might be greater than previously estimated in southeastern U.S.^31^ due to the combined effect of elevated June-July maximum temperatures which are mediated by both, climate change and ENSO condition. Therefore, we highlight the importance of developing region-specific adaptation strategies based on quantification of both, climate change and ENSO effects on month-specific climate patterns and crop yields in sensitive, to the phenomenon, regions.

## Methods

To examine the effect of climate change on maize yield and monthly weather conditions, a database including historical records of non-irrigated maize yield and monthly weather data (precipitation, average, maximum, and minimum temperature) from five locations in the southeastern U.S. was assembled (Supplementary Material Table S2 – numbers 1–5). The three locations in Alabama included data from 1971–2013, whereas Georgia locations included data from 1981–2013. To evaluate the effect of climate change on maize yield the March-September month-specific cumulative precipitation and average, maximum and minimum air temperatures were used in the analysis as predictor variables. Additionally, two indices were evaluated as explanatory variables, the Shannon diversity index- SDI^39^, and the abundant and well-distributed rainfall-AWDR index^40^. The SDI values ranges between 0–1 and values closer to 1 imply more evenly distributed rainfall in a given period, whereas larger AWDR values (no boundaries for its possible values) represent abundant and well-distributed water availability.

Because it is difficult to isolate and remove all possible non-weather factors that might affect crop yield, prior the analysis we calculated first-year differences (year-to-year changes)^3^ for the maize yields and explanatory variables to minimize the impact of other long-term factors. However, it was not possible to include all variables to a single multiple regression analysis due to severe multicollinearity issues. To identify the most appropriate set of variables, first-year differences (year-to-year changes) of the monthly average, minimum, maximum temperatures, monthly SDI and AWDR values, and monthly cumulative precipitation were regressed against first-year yield differences. The most appropriate model was the one using monthly maximum temperatures and monthly precipitation combined as explanatory variables. It was considered superior according to several statistical criteria, such as the smaller mean squared error (MSE), the larger coefficient of determination (R^2^) and adjusted R^2^, and variance inflation factor (VIF) smaller than 10^41^. Then, by multiplying the observed month-specific climate trends (the month-specific yearly linear trend from1981–2013 was independently generated by holding constant the variation in all other cells) at each location (Supplementary Material Table S1), with the regression coefficients from the relationships between first-year yield differences and weather variables (Table 1), we estimated the realized effect of individual historical month-specific climate trend on maize yields^2^,^42^ by location (Table 2).

To examine the influence of ENSO on month-specific climate conditions and maize yield, all the available years (1971–2013) were separated into three ENSO categories as characterized by the Multivariate ENSO Index (MEI) index (http://www.esrl.noaa.gov/psd/enso/mei/). It has been reported that during weak ENSO events, there is no clear Pacific/North American oscillation pattern which prevents influential energy propagation towards the North Atlantic^43^; therefore, in the analysis we considered only years with strong ENSO. As a classification rule we characterized years with December-March MEI ranks < 8 as La Niña. Years with December-March MEI ranks > 58 were characterized as El Niño. MEI rank values between 20 and 46 for the same 4 months would denote a year with Neutral phase. Years that the ENSO condition shifted significantly among short periods were not included in any ENSO phase and were characterized as undefined. From the 43 years, 7 years belonged to El Niño, 9 to La Niña, and 16 to the Neutral phase of ENSO (Supplementary Material Table 3). To further examine the influence of ENSO on parameters that influence maize yield, an additional database including monthly soil moisture content data from 2003–2013 from Blairsville, Tifton, and Calhoun (Supplementary Material Table S2 – numbers 2, 4, 6), was assembled.

## Additional Information

**How to cite this article**: Mourtzinis, S. *et al*. Climate Change and ENSO Effects on Southeastern US Climate Patterns and Maize Yield. *Sci. Rep.* **6**, 29777; doi: 10.1038/srep29777 (2016).

## Supplementary Material

Supplementary Information

## Figures and Tables

**Table 1:** Summary of statistically significant maize yield responses (P < 0.1) for a one unit increase in monthly cumulative precipitation and maximum temperature.

Region-Location	Maximum Temperature							Cumulative Precipitation						
	March	April	May	June	July	August	September	March	April	May	June	July	August	September
	**kg ha**^**−1 o**^**C** ^**−1**^							**kg ha**^**−1**^ **mm**^**−1**^						
North-Belle Mina	—	599.5	—	−1262.4	—	—	−1089.0	—	—	—	—	—	—	—
North-Blairsville	—	—	—	—	—	—	—	—	—	—	—	—	—	—
Central-Prattville	—	—	—	−735.6	—	—	608.9	—	—	14.1	8.7	15.4	—	—
Southcentral-Tifton	268.4	—	—	—	−523.5	—	—	8.0	—	18.7	14.5	−22.1	—	—
South-Fairhope	−759.7	—	—	—	—	—	—	—	−13.7	—	—	—	−10.9	—
Southeastern U.S. average	—	—	—	−610.4	−644.4	—	—	—	−9.2	—	6.3	—	—	—

The coefficients were estimated fitting linear regression models between first year differences (year-to-year changes) of yield (kg ha^−1^), of monthly maximum temperature (left), and monthly cumulative precipitation (right) generated for each of the 5 locations and each of the 7 growing season months from 1981–2013. The number in each cell is the month-specific yearly linear trend and was independently generated by holding constant the variation in all other cells. The location-specific data were aggregated to generate a southeastern US average for the same 7 months. Dashes denote statistically non-significant maize yield responses (P > 0.1).

**Table 2:** Summary of statistically significant (P < 0.1) observed maize yield trends due to the realized monthly precipitation and maximum temperature anomalies.

Region-Location		Maximum Temperature						Precipitation						
	March	April	May	June	July	August	September	March	April	May	June	July	August	September
	**kg ha**^**−1**^ **year**^**−1**^													
North-Belle Mina	—	36	—	−88.8	—	—	−59.3	—	—	—	—	—	—	—
North-Blairsville	—	—	—	—	—	—	—	—	—	—	—	—	—	—
Central-Prattville	—	—	—	−34.2	—	—	11.2	—	—	−11.9	−12.1	−8.6	—	—
Southcentral-Tifton	3.2	—	—	—	10.3	—	—	−10	—	−13.2	22.9	−14.7	—	—
South- Fairhope	−35	—	—	—	—	—	—	—	2.0	—	—	—	−2.1	—
Southeastern U.S. average	—	—	—	−18.6	0.2	—	—	—	4.1	—	−2.8	—	—	—

The estimates were calculated by multiplying the observed monthly precipitation and maximum temperature trends (mediated by climate change) that are documented in Table S2 and the estimated potential impacts on yield that are documented in Table 2 for each of the 5 locations over the past 33 years (1981–2013). The location-specific data were aggregated to generate a southeastern-US average for each of the 7 months. Dashes denote statistically non-significant observed maize yield trends (P > 0.1).
