# Supplementary material for: Climate Change and ENSO Effects on Southeastern US Climate Patterns and Maize Yield
Source: Sci Rep. 2016 Jul 19;6:29777. doi: 10.1038/srep29777 (PMC4949459; doi:10.1038/srep29777)
Supplement: Supplementary Information [file srep29777-s1.doc]

**Climate Change and ENSO Effects on Southeastern US Climate Patterns and Maize Yield**

Spyridon Mourtzinis*^1^, Brenda V. Ortiz^2^, and Damianos Damianidis^2^

1) Department of Agronomy, University of Wisconsin-Madison, Madison WI 53706.

2) Department of Crop, Soils & Environmental Sciences, Auburn University, 201 Funchess Hall, Auburn, AL 36849.

*Corresponding author: [mourtzinis@wisc.edu](mailto:mourtzinis@wisc.edu)

Table S1. Summary of observed monthly cumulative precipitation and maximum temperature anomalies within each location and aggregated into southeastern U.S. average values (1981-2013). The number in each cell is the month-specific yearly linear trend (1981-2013).

|  | Maximum Temperature trend | | | | | | | Cumulative Precipitation trend | | | | | | |
| --- | --- | --- | --- | --- | --- | --- | --- | --- | --- | --- | --- | --- | --- | --- |
| Region-Location | March | April | May | June | July | August | September | March | April | May | June | July | August | September |
|  | ^o^C year^-1^ | | | | | | | mm year^-1^ | | | | | | |
| North-Belle Mina | 0.042 | 0.060 | 0.036 | 0.070 | 0.037 | 0.062 | 0.054 | -0.533 | -0.269 | -0.188 | -2.032 | 1.411 | -1.642 | -0.082 |
| North-Blairsville | 0.032 | 0.067 | 0.016 | -0.005 | -0.024 | 0.024 | 0.029 | 0.222 | 0.583 | -0.554 | 0.339 | 0.951 | 0.935 | 1.157 |
| Central-Prattville | 0.037 | 0.046 | 0.030 | 0.046 | 0.014 | 0.029 | 0.018 | -1.159 | -2.322 | -0.841 | -1.395 | -0.557 | -0.023 | 0.195 |
| Southcentral-Tifton | 0.012 | 0.011 | -0.006 | 0.0001 | -0.020 | 0.004 | -0.006 | -1.260 | -0.073 | -0.705 | 1.582 | 0.664 | 0.720 | 1.027 |
| South- Fairhope | 0.046 | 0.038 | 0.036 | 0.046 | 0.010 | 0.031 | 0.029 | -2.388 | -0.142 | -0.511 | -0.743 | 2.562 | 0.187 | 2.873 |
| Southeastern U.S. average | 0.034 | 0.044 | 0.022 | 0.030 | 0.0001 | 0.027 | 0.030 | -1.023 | -0.445 | -0.560 | -0.445 | 1.142 | -0.038 | 0.813 |

Table S2. Information of the locations that data was retrieved and analyzed.

| Number | Region-Location | State | Coordinates | Soil type |
| --- | --- | --- | --- | --- |
| 1 | North-Belle Mina | AL | 34º40’23.59” N, 86º53’12.95” W | Silt loam |
| 2 | North-Blairsville | GA | 34º52’34.32” N, 83º57’29.67” W | Clay loam |
| 3 | Central-Prattville | AL | 32º27’50.49” N, 86º27’34.91” W | Fine sandy loam |
| 4 | Southcentral-Tifton | GA | 31º27’01.67” N, 83º30’30.59” W | Loamy sand |
| 5 | South- Fairhope | AL | 30º31’22.70” N, 87º54’11.97” W | Fine sandy loam |
| 6 | North-Calhoun | GA | 34º30’09.31” N, 84º57’03.80” W | Clay loam |

Table S3. Classification of years 1971-2013 to El Niño/Southern Oscillation (ENSO) phases according to the Multivariate ENSO Index (MEI).

| ENSO phase | | | |
| --- | --- | --- | --- |
| La Niña | Neutral | El Niño | Undefined |
| Year | | | |
| 1971 | 1972 | 1973 | 1980 |
| 1974 | 1977 | 1983 | 1985 |
| 1975 | 1978 | 1987 | 1988 |
| 1976 | 1979 | 1992 | 1990 |
| 1989 | 1981 | 1995 | 1993 |
| 1999 | 1982 | 2003 | 1994 |
| 2000 | 1984 | 2010 | 1998 |
| 2008 | 1986 |  | 2005 |
| 2011 | 1991 |  | 2007 |
|  | 1996 |  | 2009 |
|  | 1997 |  | 2012 |
|  | 2001 |  |  |
|  | 2002 |  |  |
|  | 2004 |  |  |
|  | 2006 |  |  |
|  | 2013 |  |  |


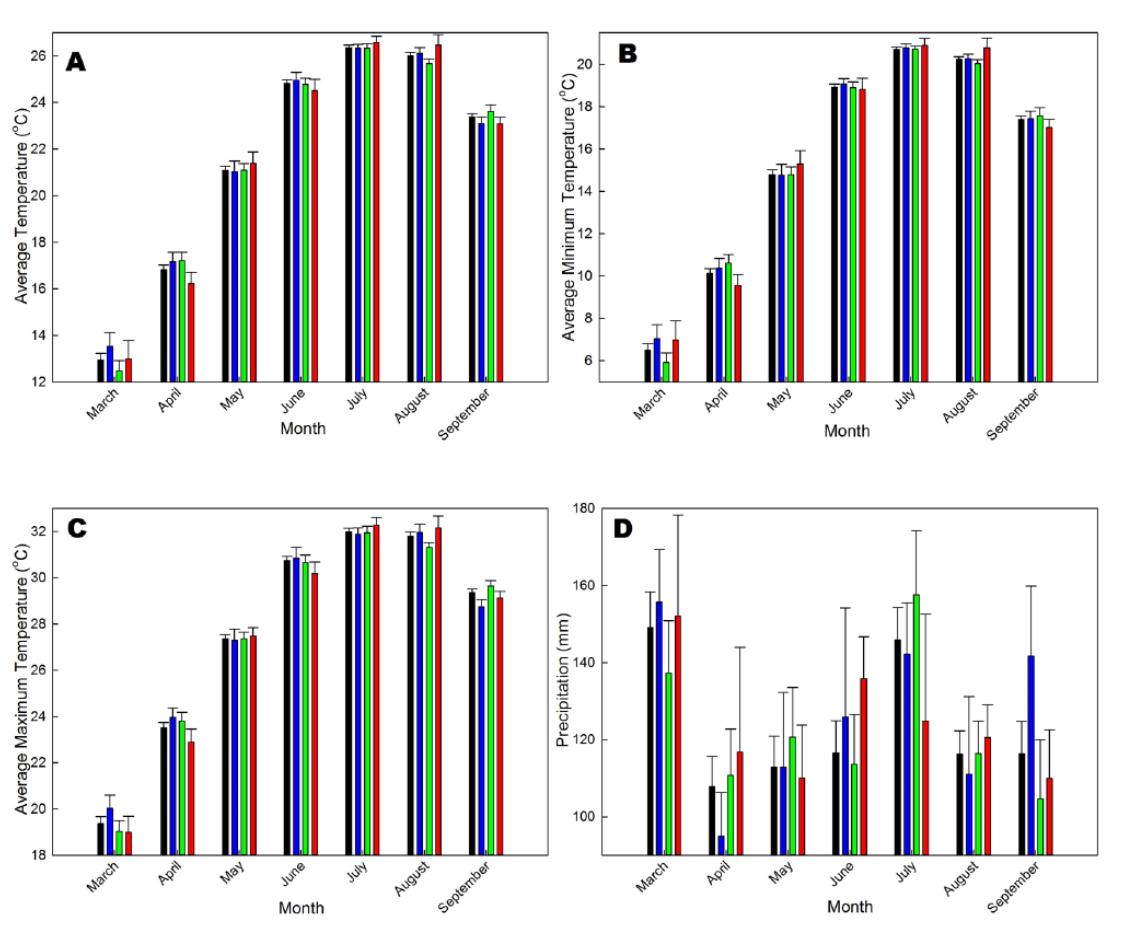


**Figure S1A-D. | Month-specific average temperature (A), minimum temperature (B), maximum temperature (C) and cumulative precipitation (D) averaged across locations (1971-2013) by ENSO phase.** Bars in black color represent the 43-year average values. Bars in blue color represent the average values of the La Niña years. Bars in green color represent the average values of the Neutral years. Bars in red color represent the average values of the El Niño years. Standard errors represent the standard error of the mean.


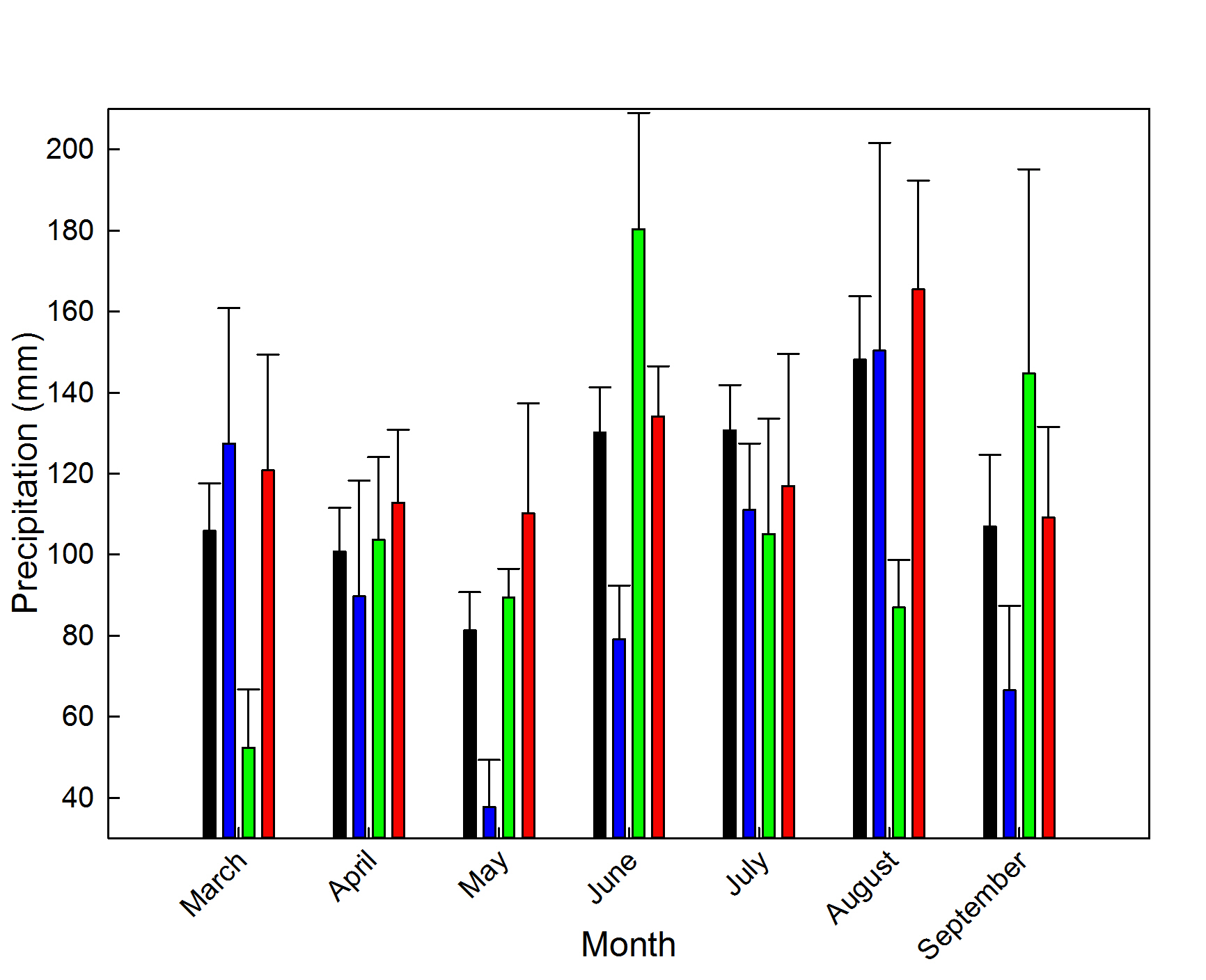


**Figure S2. | Month-specific cumulative precipitation (mm) averaged across Blairsville, Tifton, and Calhoun in GA (2003-2013) by ENSO phase.** Bars in black color represent the 11-year average values. Bars in blue color represent the average values of the La Niña years. Bars in green color represent the average values of the Neutral years. Bars in red color represent the average values of the El Niño years. Standard errors represent the standard error of the mean.
